# Modeling antibody dynamics following herpes zoster indicates that higher varicella-zoster virus viremia generates more VZV-specific antibodies

**DOI:** 10.3389/fimmu.2023.1104605

**Published:** 2023-02-16

**Authors:** Hajar Besbassi, Irene Garcia-Fogeda, Mark Quinlivan, Judy Breuer, Steven Abrams, Niel Hens, Benson Ogunjimi, Philippe Beutels

**Affiliations:** ^1^ Centre for Health Economics Research and Modeling Infectious Diseases (CHERMID), Vaccine and Infectious Disease Institute (VAXINFECTIO), University of Antwerp, Antwerp, Belgium; ^2^ Antwerp Unit for Data Analysis and Computation in Immunology and Sequencing (AUDACIS), University of Antwerp, Antwerp, Belgium; ^3^ Antwerp Center for Translational Immunology and Virology (ACTIV), Vaccine and Infectious Disease Institute (VAXINFECTIO), University of Antwerp, Antwerp, Belgium; ^4^ Division of Infection and Immunity, University College London, London, United Kingdom; ^5^ Global Health Institute (GHI), Family Medicine and Population Health (FAMPOP), University of Antwerp, Antwerp, Belgium; ^6^ Data Science Institute (DSI), Interuniversity Institute for Biostatistics and Statistical Bioinformatics (I-BioStat), UHasselt, Hasselt, Belgium; ^7^ Department of Paediatrics, Antwerp University Hospital, Edegem, Belgium

**Keywords:** varicella zoster virus, herpes zoster, antibody levels, ordinary differential equations, nonlinear mixed-effects models, mathematical modeling

## Abstract

**Introduction:**

Studying antibody dynamics following re-exposure to infection and/or vaccination is crucial for a better understanding of fundamental immunological processes, vaccine development, and health policy research.

**Methods:**

We adopted a nonlinear mixed modeling approach based on ordinary differential equations (ODE) to characterize varicella-zoster virus specific antibody dynamics during and after clinical herpes zoster. Our ODEs models convert underlying immunological processes into mathematical formulations, allowing for testable data analysis. In order to cope with inter- and intra-individual variability, mixed models include population-averaged parameters (fixed effects) and individual-specific parameters (random effects). We explored the use of various ODE-based nonlinear mixed models to describe longitudinally collected markers of immunological response in 61 herpes zoster patients.

**Results:**

Starting from a general formulation of such models, we study different plausible processes underlying observed antibody titer concentrations over time, including various individual-specific parameters. Among the converged models, the best fitting and most parsimonious model implies that once Varicella-zoster virus (VZV) reactivation is clinically apparent (i.e., Herpes-zoster (HZ) can be diagnosed), short-living and long-living antibody secreting cells (SASC and LASC, respectively) will not expand anymore. Additionally, we investigated the relationship between age and viral load on SASC using a covariate model to gain a deeper understanding of the population’s characteristics.

**Conclusion:**

The results of this study provide crucial and unique insights that can aid in improving our understanding of VZV antibody dynamics and in making more accurate projections regarding the potential impact of vaccines.

## 1 Introduction

Varicella zoster virus (VZV) is a neurotropic double-stranded DNA virus of the Herpesviridae family and the subfamily alpha-herpesvirinae, which is only hosted by humans. After infection of the nasopharyngeal lymphoid tissue, the virus spreads to the regional lymph nodes and induces viremia before infecting the skin and causing the typical rash (1). After retrograde transmission from the skin to the sensory neurons, this is followed by a latent phase in the sensory neurons in the posterior horn of the spinal cord. Clinical (and subclinical) reactivation leads to herpes zoster (shingles) and is promoted by several factors such as decreased T-cell immunity and stress.

Many studies have focused on the characterization of the secondary immune response following human vaccination or using animal challenge studies. However, the study of secondary immune responses after natural re-exposure in humans has received far less attention. An exceptional series of observational studies has focused on immune responses following exogenous re-exposure to VZV (2–4).

The application of mathematical models to describe immune dynamics, which lie behind longitudinal immunogenicity data post vaccination, has provided us with new avenues to test the validity of theoretical biological model constructs describing the dynamics of antibody-secreting cells following re-exposure, or to propose new theoretical models.

For instance, long-term antibody decay dynamics following Hepatitis A Virus vaccination have been modeled longitudinally (5) and this analysis supported the imprinted lifespan model, formulated by Amanna and Slifka (6), assuming the presence of two types of antibody producing plasma cells. However, until now such analyses have not yet been performed on data concerning exogenous re-exposure in humans or for antibody dynamics during ongoing endogenous antigen exposure.

Here, we applied mathematical models to describe antibody titer concentrations collected longitudinally after herpes zoster onset, thereby representing endogenous re-exposure to VZV. In previous research (7), the use of this framework has proven to be insightful with regard to B- and T-cell dynamics following VZV vaccination. A system of ordinary differential equations (ODEs) was formulated to model the dynamics of both B- and T-cells and this system was used to draw conclusions with regard to the underlying immunological processes. For example, Keersmaekers and colleagues (7) found that the difference in T-cell dynamics following two different types of VZV vaccines was partially due to the difference in the proliferation rate of these T-cells induced by vaccination. In addition to Keersmaekers et al. (7), other researchers have also used mathematical modeling based on ODEs to study immune responses in humans. Pasin et al. (8) used ODEs to study the effects of IL-7 injections on T-cell dynamics in HIV-infected patients. Picat et al. (9) used ODEs to study the effects of IL-7 injections on T-cell dynamics in HIV-infected patients. Picat et al. (9) used machine learning to study chronic immune T-cell activation in successfully treated HIV patients. Both studies used mathematical modeling and data analysis techniques to investigate immune responses in humans and have potential implications for the treatment of HIV infection and other immune-related conditions.

Here, a similar approach was used to gather further insight into the immunological processes underlying the secondary immune response after natural endogenous re-exposure elicited by VZV reactivation. We developed multiple models and applied them to observational data. The results of our approach are described in detail in Section 3. A further discussion about the key findings, the advantages and disadvantages of our approach and avenues for future research are presented in Section 4.

## 2 Materials and methods

### 2.1 Data

**Figure 1:**
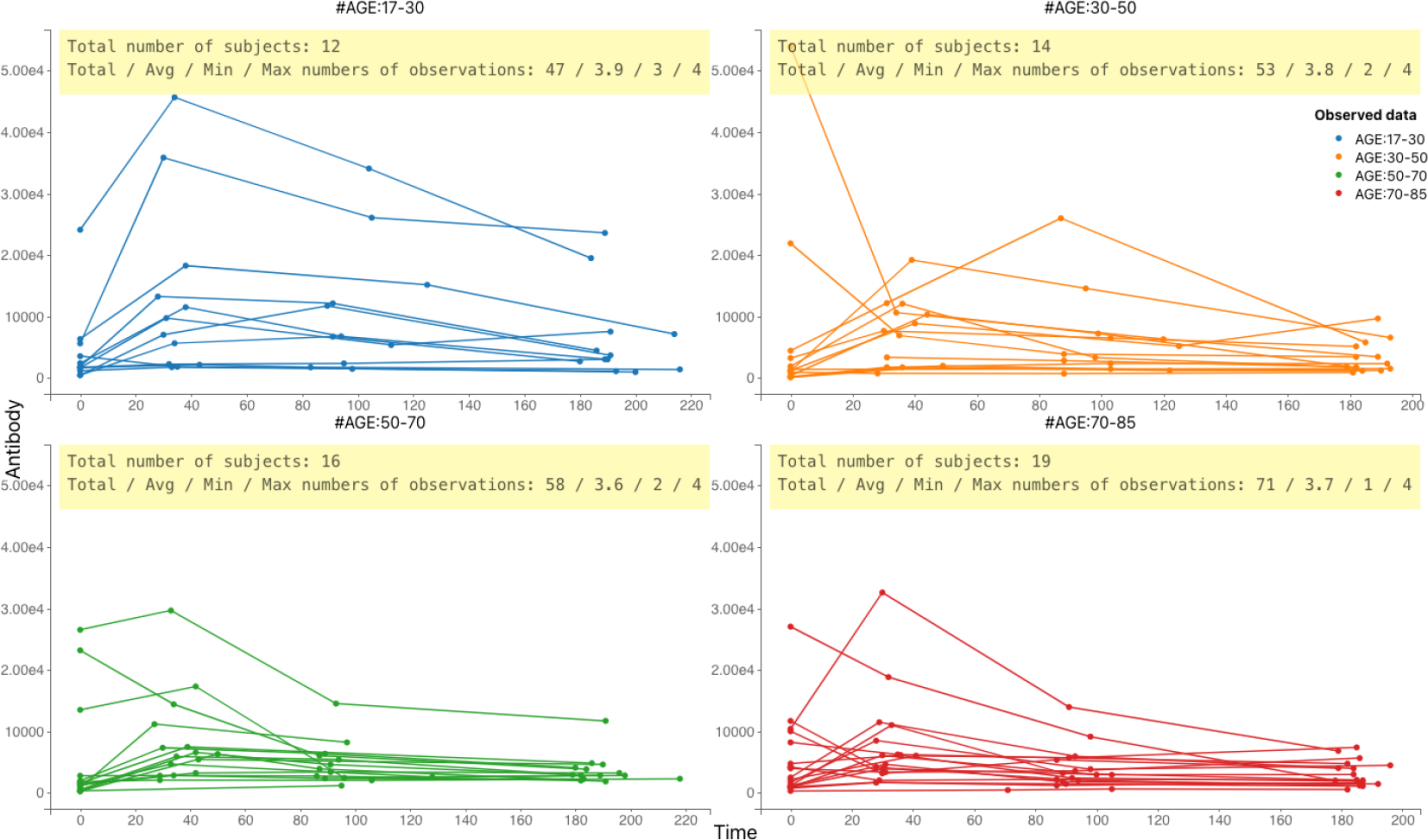
Individual-specific VZV IgG antibody titer concentration (expressed in mIU/ml) profiles by time (in days) since symptom onset in 61 herpes zoster patients. Data are shown per age group.

We used data derived from a study conducted in the UK (10) in which a total of sixty-one herpes zoster (HZ) patients was recruited at onset of HZ rash symptoms. VZV Immunoglobulin G (IgG) antibody titer concentrations (mIU/ml) were determined at symptom onset, and one month, three months and six months after symptom onset. The timing of the assessment of the IgG antibody titer levels is not equal across all individuals in the sample. The corresponding longitudinal profiles are depicted in **Figure 1**. For most individuals, antibody levels rise quickly after the onset of HZ symptoms, followed by a decrease at a slower rate. Background information comprised participants’ age (range: 17-85 years), sex (45% females), and whether they used antivirals during the study (67% of the patients did), and we used a single time point of viral load at the commensal point. This sample was collected at baseline and was the only one taken into account in order to reduce the complexity of the calculation. We used this information to study the influence of these factors on the antibody dynamics.

### 2.2 Mathematical models

To describe the antibody dynamics, we utilized systems of (nonlinear) ODEs. A systematic strategy was used to fit and assess the performance of multiple models to identify the ones that describe the data best while offering enough biological meaning. **Appendix A** contains a comprehensive summary of the systems of ODE associated with the different models. The reasoning for the use of these ODE systems to describe antibody dynamics is presented in the subsections that follow.

#### 2.2.1 Antibody dynamics models

We formulated different systems of nonlinear ODEs to model the observed antibody dynamics. We assumed that the incubation period is the same for all individuals and we take the value proposed in the literature. We assumed antibody levels to change over time due to a proliferation function (*f*_1_) and a decay function (*f*_2_). In all models, we assumed that proliferation depended on the number of antibody secreting cells (ASC, e.g., plasma cells), and decay on the number of antibodies (AB). For ease of presentation, we suppress the time dependence of the number of AB and the number of ASC from the notations below. Hence, we can express the progression of the number of antibodies in the following general differential equation:


(*i*) $$\frac{dAB}{dt}=f_{1}\left(\text{ASC}\right)-f_{2}\left(\text{AB}\right)$$

Next, we describe the number of ASC using a second differential equation. We assume that proliferation of ASC occurs according to a function *g*_1_, during a time period [0, *h*] after which no new ASC will be generated. ASC decay is assumed to occur at all-time points according to a decay function *g*_2_. This leads to the second differential equation:


(*ii*) $$\frac{dASC}{dt}=g_{1}\left(\text{ASC}\right)I_{t\leq h}-g_{2}\left(\text{ASC}\right)$$

where *I_t≤h_* represents an indicator function that takes value one if *t ≤ h*, and is equal to zero otherwise.

In all our proposed models, antibody decay is assumed to be proportional to the number of antibodies and can therefore be written as: *f*_2_ (AB) = *u_AB_* × AB, with *u_AB_* denoting a time-invariant decay rate (i.e., implying exponential decay). Similarly, the antibody production is assumed to be proportional to the number of ASC, and thus *f*_1_ (ASC) = *p_AB_* × ASC, where *p_AB_* is the constant antibody production rate by ASC. Equations (1) and (2) can be combined as follows:


(*iii*) $$\{\begin{array}{l} \frac{dAB}{dt}=p_{AB}\times \text{ASC}-u_{AB}\times \text{AB}\\ \frac{dASC}{dt}=g_{1}\left(\text{ASC}\right)I_{t\leq h}-g_{2}\left(\text{ASC}\right), \end{array}$$

where *AB*_0_ = *AB*(0) and *ASC*_0_ = *ASC*(0) are the antibody and plasma cell counts at time 0 (days), respectively. We can further divide the population of ASC into two sub-populations: one with a short lifespan and the other with a long lifespan (11, 12). Short-living antibody secreting cells (SASC) can be interpreted as B-cells (whether or not including plasmablasts). Long-living antibody secreting cells (LASC) can be seen as plasma cells, which can reside in the bone marrow with a lifespan of many years (13, 14).

Models (4), (5) and (6) treat the population of ASC as a single population without discriminating between short- and long-living plasma cells, while models (7), (8) and (9) look at the aforementioned sub-populations separately. by the choice of the proliferation function *g*_1_(ASC).

In model (4), we assume that ASC expansion occurs with the constant function *g*_1_(ASC) = *p_ASC_* during time period [0, *h*]. In model (5), expansion happens proportional to the number of ASC, *g*_1_(ASC) = *p_ASC_* × ASC. Model (6) assumes no proliferation *g*_1_(ASC) = 0 and thus the number of ASC monotonously decreases over time starting from the initial level *ASC*_0_. We can interpret this as proliferation that can be neglected over a short time period [0, *h*].

Similarly, we defined models (7), (8) and (9), explicitly distinguishing between LASC and SASC. First of all, in order to limit the number of model parameters, we assume that the number of LASC remains constant over time, i.e., *d*LASC / *dt* = 0. Moreover, we assume no SASC at time 0, i.e., SASC(0) = 0 and decay of SASC following *g*_2_(SASC) = *u_SASC_* × SASC.

Models (7) to (9) rely on the use of different proliferation functions for SASC. More specifically, in model (7) a constant expansion *g*_1_(SASC) = *p_SASC_* is assumed, in model (8) a proportional expansion *g*_1_(SASC) = *p_SASC_* × SASC is considered and finally in model (9) no expansion *g*_1_(SASC) = 0 is presumed.

### 2.3 Statistical modeling

#### 2.3.1 Nonlinear mixed models

We can now formulate nonlinear mixed models based on the dynamic models described in the previous subsection. The nonlinear mixed effects model implemented is defined as:


$$y_{ij}=f\left(t_{ij},\psi_{i}\right)+g\left(f\left(t_{ij},\psi_{i}\right),\xi \right)\epsilon_{ij},\;1\leq i\leq N,\;1\leq j\leq n_{i}$$

where *y*_*ij*_∈*ℝ* is the *j*th observation in the *i*th subject, *Ψ_i_* is the parameter vector of the structural model ***f*** for individual *i*. The residual error model is defined by the function ***g*** of the structural model *f* and an additional vector of parameters *ξ*. The residual errors (*ε_ij_*) are standard Gaussian random variables (mean 0 and standard deviation 1). In this case, it is clear that *f*(*t*_*ij*_,*ψ*_*i*_) and *g*(*f*(*t*_*ij*_,*ψ*_*i*_),*ξ*) are the conditional mean and standard deviation of *y_ij_*, *E*(*y*_*ij*_|*ψ*_*i*_)=*f*(*t*_*ij*_,*ψ*_*i*_) and *sd*(*y*_*ij*_|*ψ*_*i*_)=*g*(*f*(*t*_*ij*_,*ψ*_*i*_),*xi*) .

The assumption that the distribution of any observation *y_ij_* is symmetrical around its predicted value is a very strong one. In order to achieve that, we may want to transform the data to make it more symmetric around its (transformed) predicted value. An extension of the statistical model to include a Log-normal distribution was therefore proposed as transformation since all observations are


$$\text{u}\left(y_{ij}\right)=\text{u}\left(\text{f}\left(t_{ij},\psi_{i}\right)\right)+\text{g}\left(\text{u}\left(\text{f}\left(t_{ij},\psi_{i}\right)\right),\xi \right).$$

In mixed models, both fixed and random effects are included. Consider a parameter *P_pop_* (e.g., the decay rate of antibodies) which is constant across different individuals. Such a parameter can be interpreted as a population(-averaged) parameter, representing the average value across all individuals, while the use of random effects leads to an individual-specific interpretation of parameters and a description of possible variation between individuals. An individual-specific model parameter *P_i_* can be written as *P*_*i*_=*u*_*i*_×*P*_*pop*_ , where *P_pop_* is the so-called population parameter and *u_i_* is an individual-specific random effect. In this paper, we will assume that *u_i_* follows a log-normal distribution with a unit mean (to ensure identifiability and the aforementioned interpretation for *P_pop_*) and variance *ω* ^2^.

Categorical variables, such as sex or the use of antivirals, can be taken into account by adding a dummy variable with an additional parameter *β_j_* describing how the parameter of group *j* deviates from the reference group.

For example, if we consider sex as a categorical variable assuming that the population values of antibodies are different for male and female, we implemented the following model: *log*(*A*_*i*_)=*log*(*A*_*pop*_)+*β*_*A*_1_*sex*_*i*_=*F*_+*η*_*A*,*i*_, where 1_*sex*_*i*_=*F*_ if individual *i* is a female and 0 otherwise. Then, *A_pop_* is the population antibody for males while *A*_*pop*_ *e* ^*β*_*A*_^ the population antibody for females. This allows investigating which particular parameter of the structural model (e.g., proliferation of antibodies, decay of antibody secreting cells,…) leads to the differences observed between different groups (e.g., male vs. female individuals). Dependency can be introduced between individual parameters by assuming that the random effects *η_i_* are not independent.

The model parameters were estimated using the Monolix software ^©^Lixoft (2021 version) (15). The estimation of the population parameters was achieved by a two-step algorithm with 10^6^ + 10^5^ iterations to assess convergence. This algorithm consists of a built in stochastic approximation of the standard expectation maximization algorithm (SAEM) with simulated annealing, combined with a Markov Chain Monte Carlo (MCMC) procedure which replaces the simulation step of the SAEM algorithm (16). Next, importance sampling was used to determine the log-likelihood per model, in which a fixed *t*-distribution is assumed with 5 degrees of freedom. The models were assessed using the Akaike Information Criterion (AIC) (17).

#### 2.3.2 Inference and model selection

For the antibody data set, the following procedure was used for comparing and selecting the most suitable biologically plausible model to describe the data.

In order to compare the different models and select the most suitable and biologically plausible model to describe the antibody data, we deployed the following procedure. First of all, for each model, the model parameters were estimated using the Monolix software. In case of SAEM-MCMC convergence, we used the AIC (17) to compare the different models resulting in a list of six models to be compared in the next step. Models with poor SAEM convergence, likely because of abundant model complexity, were discarded. Subsequently, the model with the lowest AIC value on the list was selected as the first candidate model. We performed a non-parametric bootstrap with 1000 bootstrap samples to investigate model stability. If the bootstrap did not converge well, we excluded the model from the list of candidate models. Since a sequential approach based on the candidate models with the lowest AIC values was used, the need to perform bootstraps for all candidate models was avoided, in order to decrease the number of computations. It was found that for a bootstrap, 63%-77% of the samples had proper SAEM convergence. For this reason, the criterion for good bootstrap convergence was defined as having at least 65% of bootstrap samples with proper SAEM convergence. Finally, a sensitivity analysis of the (converging) candidate model’s bootstrap results was performed to determine whether the presence or absence of specific profiles of individuals in the bootstrap samples influenced model convergence. For example, if a single participant’s profile appeared more frequently in a non-converging dataset, a new bootstrap was run, excluding the specific participant’s profile. Again, if the bootstrap convergence was poor, the candidate model was rejected. If convergence remained robust enough, the candidate model was chosen as the final model. (for more details see **Appendixes C** and **D**).

## 3 Results

### 3.1 Antibody dynamics

In our mathematical models, we combined ordinary differential equations with a statistical mixed model approach. ODEs allow for the translation of specific biological processes into a testable mathematical framework. These models, and the differences between the models we tested, are explained in more detail in Section 2.2.

**Table 1** displays lists of all models, along with the corresponding information criteria.

**Table 1:** Model comparison: Estimated information criteria and model performance.

Model	Model	BIC	BICc	−2×Log Likelihood	Number of parameters
Model 1	4266.62	4298.28	4308.86	4236.62	7
Model 2	4619.51	4651.17	4661.75	4589.5	7
Model 3	4259.74	4282.96	4290.9	4237.74	5
Model 4	4244.92	4280.8	4292.71	4210.91	8
Model 5	4247.61	4283.49	4295.39	4213.61	8
Model 6	4243.84	4275.5	4286.39	4213.84	7

Comparison of Akaike Information Criteria (AIC), Bayesian Information Criteria (BIC) and Corrected Bayesian Information Criteria (BICc) values for different models, along with the estimated -2× Log-Likelihood and number of parameters fit. Model 6 is selected as the final candidate model due to its lowest AIC, BIC, and BICc values and high convergence rate.

Based on the results presented in **Table 1**, we can see that model 6 has the lowest AIC, BIC, and BICc values among all the models considered. Additionally, the results from the bootstrap analysis show that model 6 has the highest convergence rate of 96%. These results suggest that model 6 is the most robust and reliable model for explaining the experimental VZV IgG data.

Model 9 assumes an underlying structure of antibody-secreting cells (ASC) that differentiate between short-lived antibody-secreting cells (SASC) and long-lived antibody-secreting cells (LASC). The number of LASC is assumed to remain constant over time, while the number of SASC decays at a rate of *u_SASC_* without proliferation. Antibody dynamics are then assumed to be proportional to the number of LASC (*p_ABL_*) and the number of SASC (*p_ABS_*), with an antibody decay rate of *u_AB_*. Each parameter is considered to have a random effect, i.e., a population component and an individual component.

Therefore, Model 9 suggests that the experimental VZV IgG data measured after VZV reactivation had already resulted in SASC expansion, and that the ASC expansion had already stopped when VZV reactivation became clinically significant. This finding has important implications for understanding the immune response to VZV and could potentially inform vaccine development efforts.

**Figure 2:**
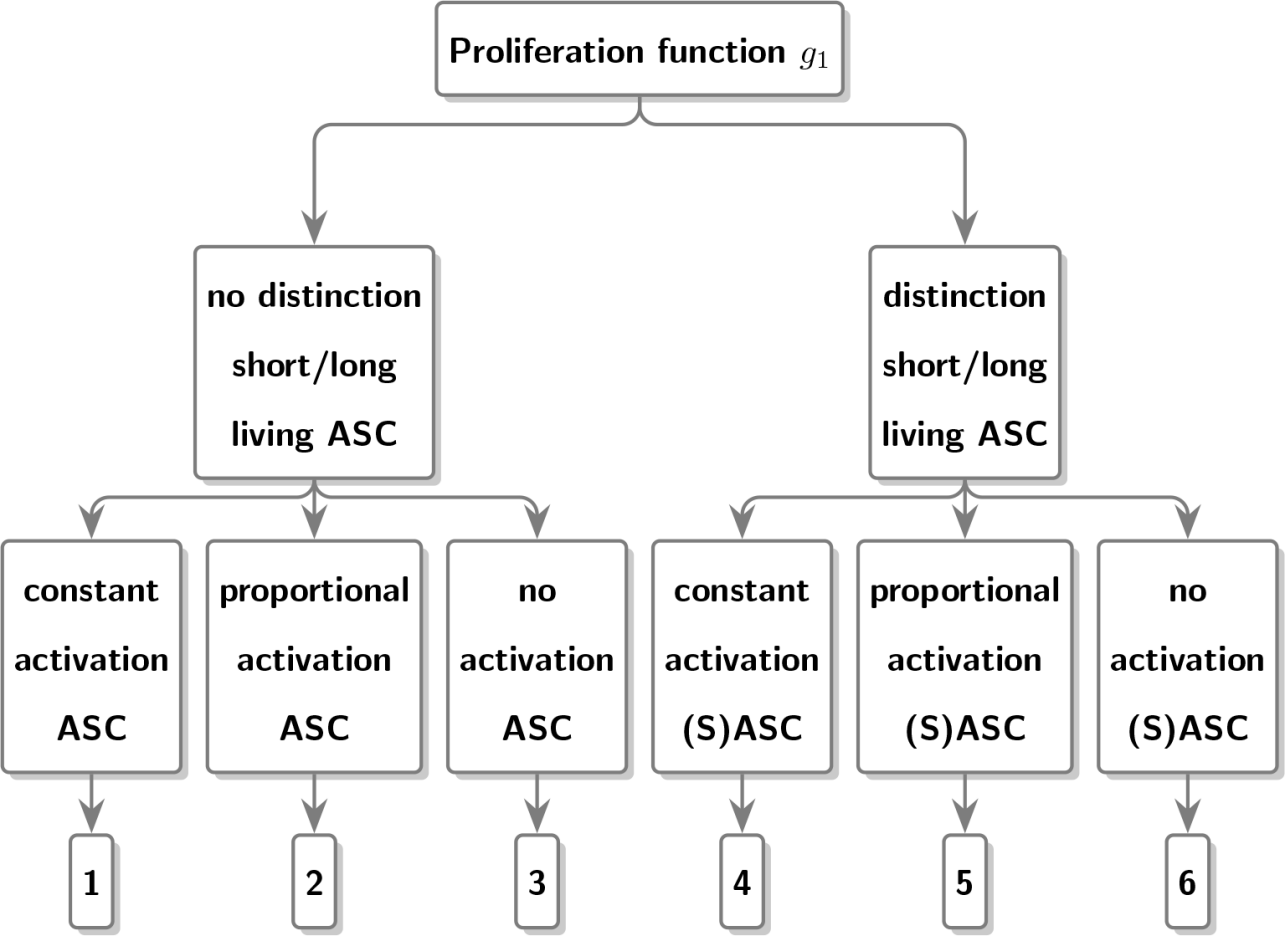
Schematic diagram depicting the different proliferation functions used to characterize antibody dynamics in the different models.

**Figure 3:**
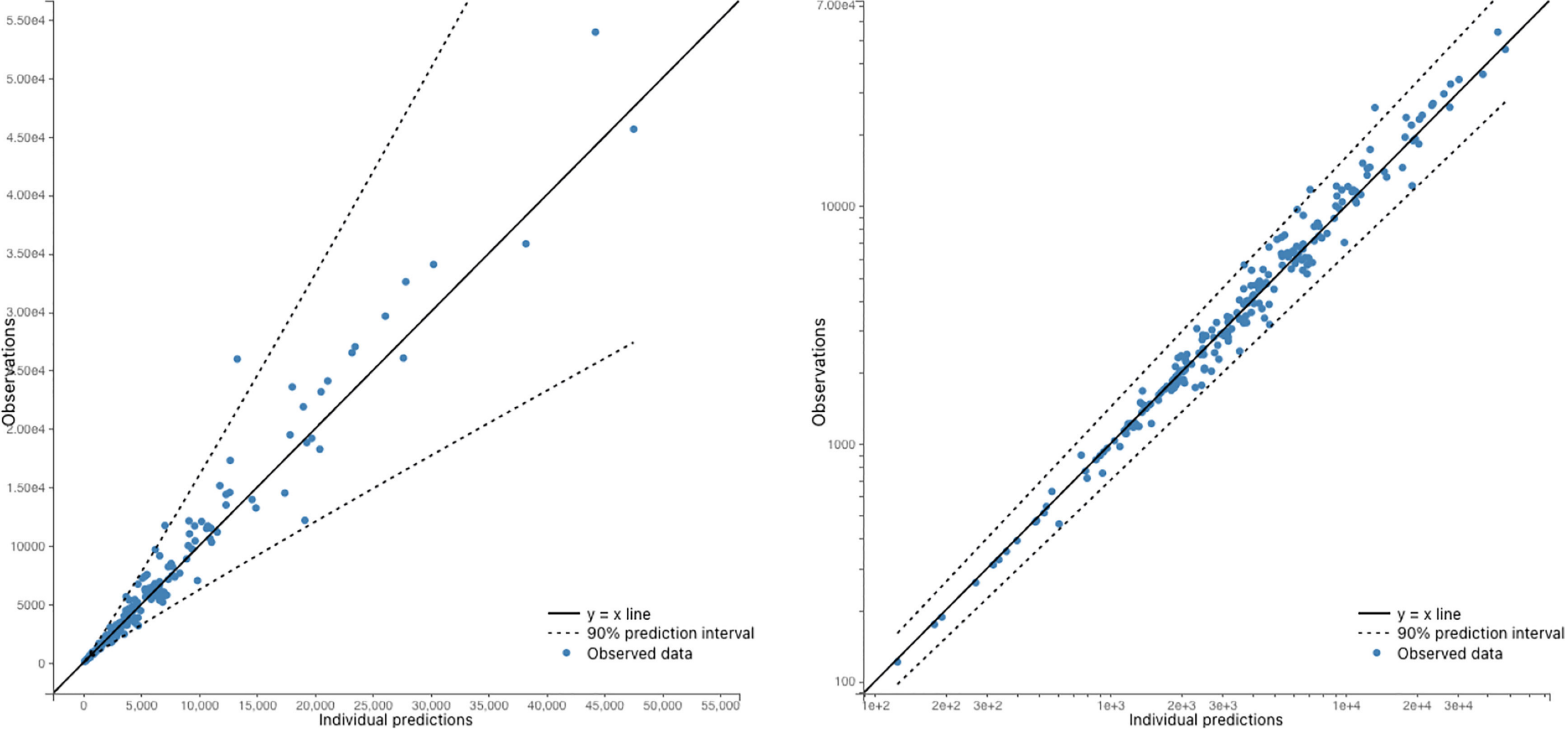
Observed antibody levels vs Model 6 predictions. The dots represent the observed data, while the y=x line represents the predicted values. The 95% predicted interval is also plotted, which shows the range within which we would expect 95% of the observations to fall. The plots are presented on both a linear scale (right) and a log-log scale (left). Overall, the figure suggests that the model predictions are in good agreement with the observed data, as the dots fall within the predicted interval for the majority of the data points.

**Figure 4:**
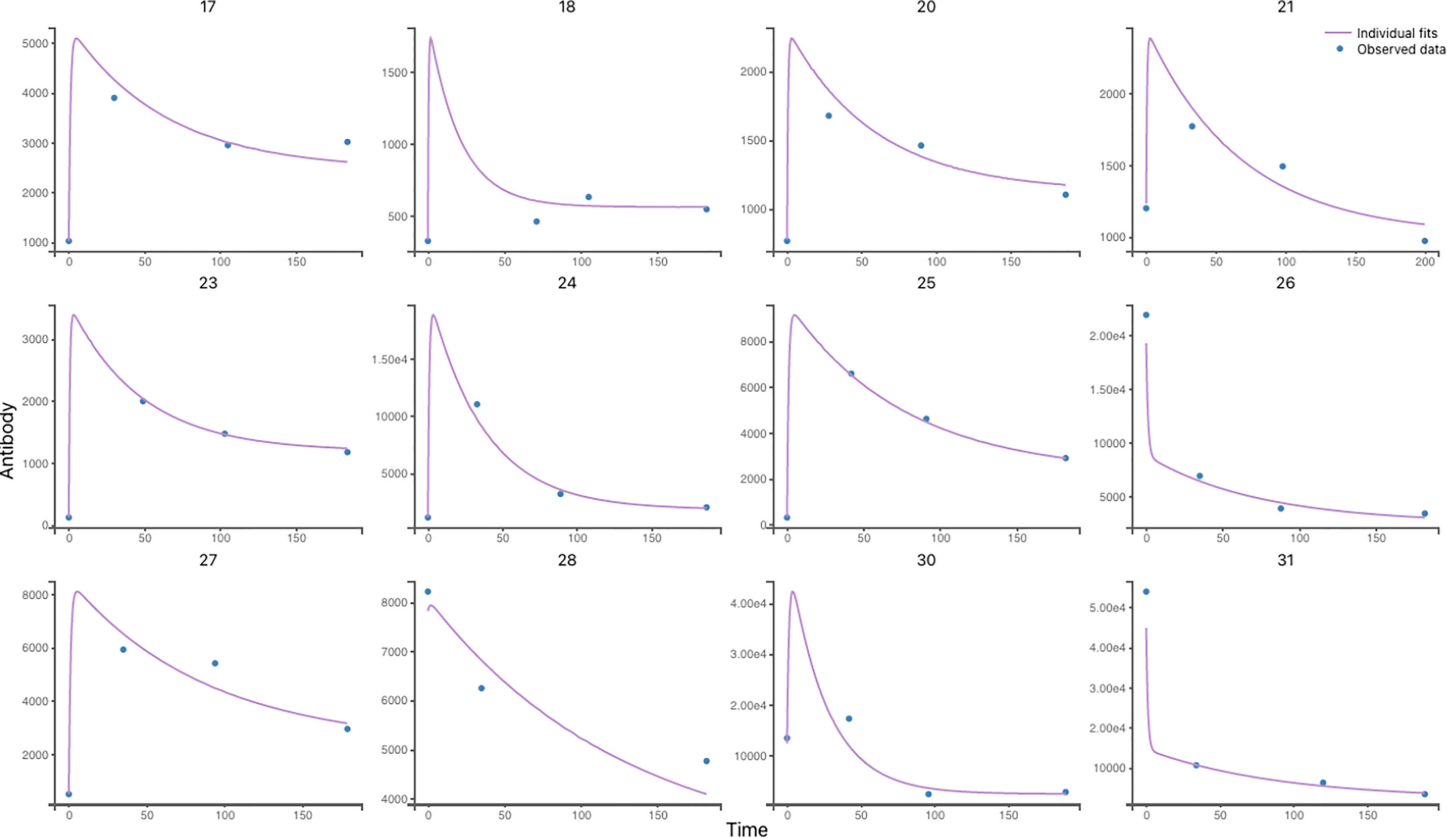
Individual antibody response to VZV Reactivation in 12 participants using Model 6. Individual predictions using individual parameters and individual variables with respect to time on a continuous grid with observed VZV antibody data overlaid are displayed in this graphic. (more details about other participants are shown in **Figure 9**).

**Figure 5:**
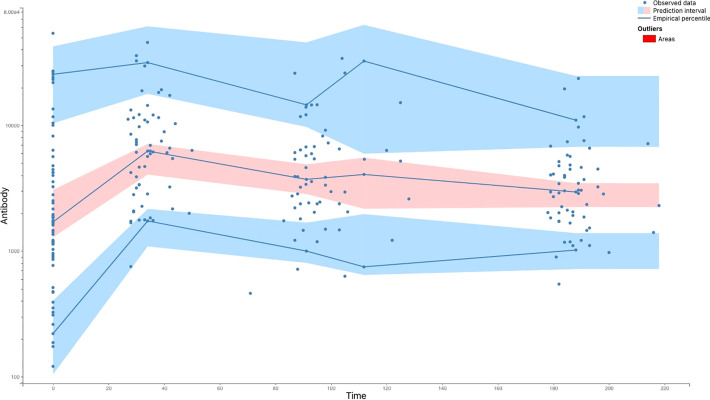
The Visual Predictive Check (VPC) comparing the results of the model-based simulations with observed data. The blue lines are empirical percentiles and summarize the observed data. The blue and pink areas are 95% prediction intervals and summarize predictions from the model 9. The observed percentiles are close to the predicted percentiles and remain within the corresponding prediction intervals.

Based on the data in **Figure 1**, we developed several ODE models, which are shown in **Figure 2**. **Figure 3** demonstrates that the observed values are within the confidence interval of the predicted values for Model 9, which we consider to be the best model. **Figure 4** shows the individual fit for 12 of the 61 participants, further validating the accuracy of Model 9 (the remaining participants can be found in **Figure S3 in the appendix D**). In **Figure 5**, we compare the simulations results obtained from our best model and the observed data using the visual predictive check (VPC). It is noticeable that the predicted percentiles are close to the observed percentiles and remain within 95prediction intervals, which highlights the accuracy of our best model.

### 3.2 Influence of correlations and covariates on model parameters

#### 3.2.1 Correlation between random effects

It is possible to introduce dependency between individual parameters by assuming that the random effects *η_i_* are not independent. This can be done by introducing linear correlation between the random effects. To test for this type of correlation, we performed Pearson’s correlation tests. In our scenario, we found a significant correlation between *η*_*AB*_0__ (the initial value of antibodies) and *η*_*μ*_*AB*__ (the decay rate of antibodies). This indicates that the distributions of these two random effects are not independent and that the correlation must be included in the model and estimated.

Once the correlation is included in the model, the random effects for *AB*_0_ and *μ_AB_* are drawn from a joint distribution rather than two independent distributions. In our scenario, including the correlation in the model resulted in an AIC of 4232.25, compared to 4243.84 for the original model. No other significant correlations were found.

#### 3.2.2 Individual parameters vs covariates

In order to understand the drivers of inter-individual variability, building a covariate model is a critical effort to explain antibody population dynamics. Many runs are usually required to find a solid covariate model (18). Several ways to automate this operation have been proposed in the past. Stepwise Covariate Modeling (SCM) is the most often utilized. We apply a new stepwise strategy based on statistical tests between individual parameters taken from their conditional distribution and covariates validated by Lixoft and published elsewhere (18).

The Conditional Sampling usage for Stepwise Approach based on Correlation testing (COSSAC) uses the information in the current model to determine which parameter-covariate relationship to test next. This technique drastically decreases the number of covariate models examined while keeping the models that improve the log-likelihood on the search path. The Pearson correlation test for continuous covariates and ANOVA for categorical covariates can be used to calculate p-values. The p-values are used to sort all of the random effect-covariate correlations, regardless of whether or not they are included in the model. Depending on the results of the correlation tests, COSSAC iterations switch between forward and backward selection. Group-specific effects on chosen parameters make it possible to investigate the influence of covariates and correlation in the selected model 9. More specifically, we investigated whether participants’ sex, age, viral load, and use of antivirals affect model parameters, as well as whether there is any correlation between random effects.

We acquired the best fitting model after testing and running all possible parameter-covariate relationships, resulting in an AIC value of 4214.14 improving the AIC value of the original model 9 by 29.7 points (see **Table 2** for the estimates of the model parameters). Since there is a significant correlation between *AB*_0_ and *u_AB_* in this model, where *AB*_0_ is the initial value of the antibodies and *u_AB_* is the decay rate allowing the correlation to be included in the model and approximated.

**Table 2:** Parameter estimates and corresponding 95% confidence intervals (CI) of final model 6.

Parameter	Estimate	95% CI	P-value
*AB*(0)	2043.49	(1446.367, 2942.913)	
*SASC*(0)	0.1227	(0.013, 0.215)	
*LASC*(0)	39.631	(20.611, 139.461)	
*pABL*	13.3669	(6.563, 45.608)	
pABS	8138.55	(10589.869, 153685.949)	
*β_pABS_* (V L)	0.4924	(0.164, 0.718)	1.32e-4
*uAB*	0.2637	(0.174, 1.066)	
*uSASC*	0.0115	(0.006,0.032)	
*βu_SASC_* (AGE)	0.9876	(0.083, 2.379)	9.41e-4
corr(*AB_0_, u_AB_*)	-0.2853	(-0.896, -0.549)	1.53e-6

AB(0), SASC(0) and LASC(0) denote the initial number of antibodies, short living and long living antibody secreting cells respectively. p_ABL_ and p_ABS_ are antibody proliferation rates, proportional to the number of antibody secreting cells. Decay of antibodies occurs at rate u_AB_, and decay of SASC at rate u_SASC_.

**Figure 6:**
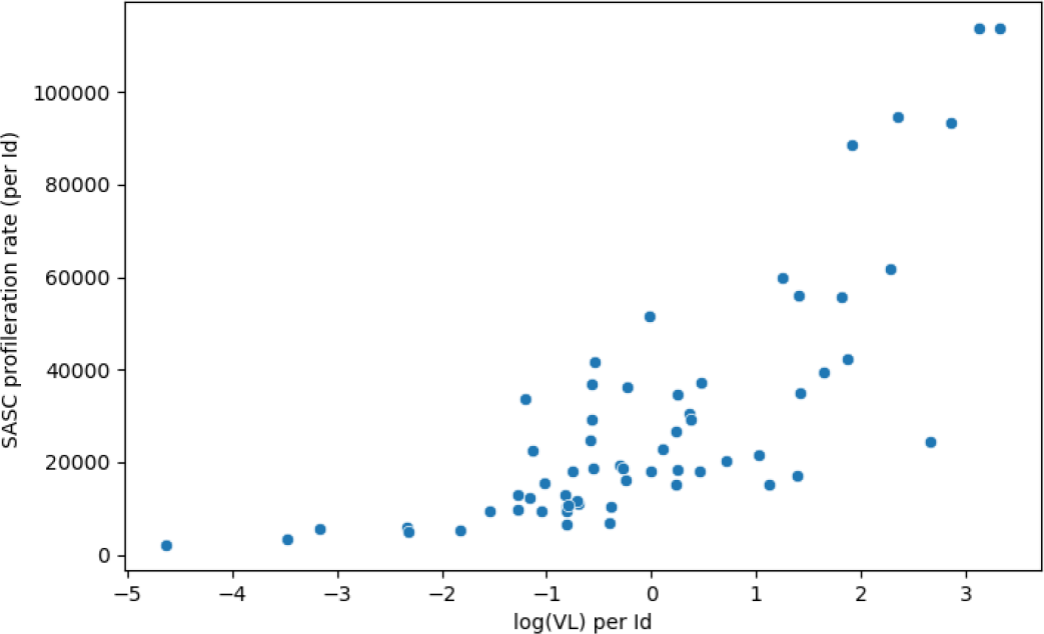
The proliferation rate of SASC increases with the presence of a higher viral load. Correlation coefficient is 0.84 and p-value of 2.97×10^-15^.

This model, on the other hand, yielded viral load and age as significant covariates. More particularly, an increasing viral load was found associated with a much higher proliferation rate *p_ABS_* (see **Figure 6**). The decay rate of SASC, *u_SASC_*, was shown to decline considerably with increasing age.

Viral load refers to the amount of virus present in a person’s body and is typically measured in terms of the concentration of virus in a sample of blood or other bodily fluid (19). High viral load can indicate a more severe infection or a greater risk of transmission to others. Age is another important factor that can influence immune function and the body’s response to infection or vaccination (20, 21). As people get older, their immune system may become less efficient at mounting a response to foreign substances, such as viruses or vaccines.

Specifically, we found that increasing viral load was associated with a higher production rate of antibodies by SASC (*p_ABS_*). This finding is supported by the study of (22), which found that increased levels of gp350-specific neutralizing activity were directly correlated with higher peripheral blood Epstein-Barr virus DNA levels during acute infectious mononucleosis. This suggests that individuals with higher viral loads may have a more pronounced immune response to varicella-zoster virus, resulting in the production of more antibodies. This has important implications for understanding the immune response to varicella-zoster virus and could potentially inform vaccine development efforts. It is worth noting, however, that while higher viral loads may lead to increased antigen exposure and potentially more need for antibody production, it is also possible that individuals with higher viral loads may have less effective antibodies and therefore require a stronger immune response to control the virus. Further research is needed to confirm and better understand these findings and the underlying mechanisms involved.

We found that sex and antiviral load did not significantly affect the results of the model. This may be due to the small sample size or the short duration of the study. Further research with larger sample sizes and longer follow-up periods would be needed to more fully examine these questions.

Additionally, we found that the decay rate of certain antibody-secreting cells was shown to decrease considerably with increasing age. This finding is supported by a previous study that demonstrated a positive correlation between VZV IgG and ageing (22). Further research is needed to confirm this finding and to better understand the potential underlying mechanisms involved.

## 4 Discussion

In this study, we combined a nonlinear mixed model approach with ordinary differential equations in order to explore the secondary immune response after re-exposure to Varicella-Zoster Virus. More specifically, we constructed a range of plausible models describing the observed VZV antibody dynamics from symptom onset up until six months.

In the most optimal model, the change of VZV-specific antibodies is proportional to the number of short living antibody secreting cells and the number of long living antibody secreting cells along with a constant decay rate of antibodies. The number of LASC remains constant over time, but our mathematical modeling predicted that the number of SASC decays according to a constant decay rate, after onset of symptoms. This prediction suggests that following VZV reactivation the number of SASC rapidly increases, at which point baseline sampling (thus after herpes zoster onset) might already occur after all SASC are generated (and without further SASC expansion, although there is ongoing VZV viremia). Indeed, the first VZV IgG antibody titers were measured at symptom onset and therefore a few days after the VZV re-exposure (i.e. VZV reactivation). At this time point, we expect that the number of antibody secreting cells has already been on the rise. For this reason, the proliferation rate of antibody secreting cells might not have been necessary in the prediction of antibodies and therefore in the best-fitting model.

Our natural re-exposure modeling contrasts with the situation in vaccination models, where the first measurement is made prior to vaccination. In that situation, the proliferation rate of antibody secreting cells is likely needed as a model parameter to obtain the candidate model for the antibody dynamics.

Our mathematical modeling of VZV reactivation has highlighted several potential interesting avenues to be studied in the future. We found that in the best fitting model both viral load and age were associated with some key parameters. In particular, our findings here support the role of viral load in increasing *p_ABS_*, the antibody production rate by SASC. Given that the viral load stimulates antibody production, this stands to reason. It implies that ongoing VZV viremia does not cause an ongoing SASC proliferation, but rather more antibody production by the initially - upon VZV reactivation - generated SASC. We also discovered that age has an effect on *u_SASC_*, the decay rate of SASC, causing it to decrease with increasing age. A variety of biological interpretations are possible; inspired by several articles (6, 23, 24), we postulate that as a participant’s age increases, memory B cells proliferate and differentiate into short-lived antibody-secreting cells, lowering the mortality rate. In other hand, we noticed that sex or antiviral of participants does not have an effect on our model.

Our findings indicate that the ODE model is more capable of simulating the nonlinear relationship between treatment effects and immunological response. Although the ODE model is more flexible and intends to include more factors or covariates in the model, it is also better at identifying the significant factors for an immunological response. It is very flexible in fitting immunological response data when combined with the nonlinear mixed-effects model. Furthermore, this method may overlook critical aspects. The ODE model is very beneficial for forecasting and simulating various biological scenarios and is biologically reasonable. One of the ramifications of this model is the significant computing effort and the demand for biological assumptions, which might be challenging to validate.

The limitations we encountered were the limited sample size, which only allowed for the analysis of models with moderate complexity, and the limitations in the frequency and timing of sampling. Future work should focus on estimating an optimal sampling schedule for subsequent modeling analysis in order to overcome these limitations.

Despite the fact that we have demonstrated through nonlinear mixed modeling using ODEs that following clinical VZV reactivation, short-lived antibody-producing cells do not expand for a long time, ongoing VZV circulation appears to cause a higher production of antibodies by these cells rather than an increase in their numbers. The primary objective of this study was to apply cutting-edge techniques to real immunology datasets, rather than to focus on the practical application of these approaches.

Indeed, instead of the more common group-wise or time-wise comparisons using standard comparative statistics, we concentrated on the benefits and potential of ODE modeling in combination with a mixed effect approach in the analysis of empirical re-exposure immunogenicity data. The methods developed in this work can now be quickly applied to relevant datasets to answer fundamental questions about the development of immune response systems.

## 5 Conclusion

Future research should aim to expand the sample size and diversify the population to increase the generalizability of the findings. Additionally, it would be valuable to investigate the effect of various interventions such as antiviral treatment and vaccination on SASC dynamics, and to assess the correlation between viral load and antibody production rate. Furthermore, it would be useful to conduct a long-term study to investigate the persistence of the immune response, and to compare our findings with other related studies in the field, in order to identify areas for further investigation and to establish the robustness of our conclusions.

In conclusion, this study has provided crucial insights into the dynamics of the immune response following varicella-zoster virus (VZV) endogenous re-exposure by using a nonlinear mixed modeling approach. Our findings indicate that once VZV reactivation is clinically apparent, the expansion of short-lived and long-lived antibody secreting cells will not occur anymore. This is a significant discovery that can aid in understanding the mechanisms underlying VZV antibody dynamics and in making more accurate projections about the potential impact of vaccines.

## Data availability statement

The original contributions presented in the study are included in the article/**Supplementary Material**. Further inquiries can be directed to the corresponding authors.

## Ethics statement

The studies involving human participants were reviewed and approved by family doctors in London between 2001 and 2003. Written informed consent to participate in this study was provided by the participants’ legal guardian/next of kin.

## Author contributions

BO contributed to the conception of the study and provided data from JB and MQ. BO and NH were involved in the design of the study. HB performed the mathematical and statistical analysis and wrote the first draft of the manuscript. All authors contributed to the article and approved the submitted version.
