# Supplementary material for: Modeling antibody dynamics following herpes zoster indicates that higher varicella-zoster virus viremia generates more VZV-specific antibodies
Source: Front Immunol. 2023 Feb 16;14:1104605. doi: 10.3389/fimmu.2023.1104605 (PMC9978810; doi:10.3389/fimmu.2023.1104605)
Supplement: Supplementary file 2 [file Presentation_1.pdf]

Appendix A. Models

The following differential equation describes the basic dynamics of the antibody population:

$$\begin{cases} \frac{dAB}{dt} = f_1(ASC) - f_2(AB), \\ \frac{dASC}{dt} = g_1(ASC) I_{t \leq h} - g_2(ASC), \end{cases}$$

$AB_0 = AB(0)$ and $ASC_0 = ASC(0)$ denotes the number of anitbodies and plasma cells at time 0 (days). In this equation, $f_1(ASC)$ describes the proliferation rate of AB depending on the number of ASC. Antiboy decay will occur throughout the time period and is represented by the function $f_2(AB)$. In the second differential equation, We assume that proliferation of ASC happens according to a function g_1, during a time period $[0, h]$ after which no new ASC will be activated. Decay happens at all time points according to a decay function g_2.

Assuming an equal number of ASC is activated during the time period $[0, h]$ leads to model 1:

$$\begin{cases} \frac{dAB}{dt} = p_{AB} \times ASC - u_{AB} \times AB, \\ \frac{dASC}{dt} = p_{ASC} \times I(t \leq h) - u_{ASC} \times ASC. \end{cases} \tag{1}$$

Model 2 does not possess the same assumption as previous model, the activation happens proportional to the number of ASC. In this case the model 2 is written as follow:

$$\begin{cases} \frac{dAB}{dt} = p_{AB} \times ASC - u_{AB} \times AB, \\ \frac{dASC}{dt} = p_{ASC} \times ASC \times I(t \leq h) - u_{ASC} \times ASC. \end{cases} \tag{2}$$

For Model 3, we suppose that no proliferation rate $g_1(ASC) = 0$. This yields to follow :

$$\begin{cases} \frac{dAB}{dt} = p_{AB} \times ASC - u_{AB} \times AB, \\ \frac{dASC}{dt} = -u_{ASC} \times ASC. \end{cases} \tag{3}$$

In a next step we distinguish the short-living plasma cells SASC from the plasma cells with long lifespan LASC. The total number of plasma cells is then equal to the sum of these two. We assume that, at baseline, only long-living and thus no short-living B-cells

are present. This model can be described as

$$\begin{cases} \frac{dAB}{dt} = f(SASC) + f(LASC) - f_2(AB), \\ \frac{dASC}{dt} = g_1(ASC)I_{t\leq h} - g_2(ASC), \\ ASC(t) = SASC(t) + LASC(t) \end{cases}$$

with $LASC$ remains constant over time ($\frac{dLASC}{dt} = 0$) and $SASC(0) = 0$. First, in model 4 we assume a constant proliferation rate p_{SASC}, which is leads to :

$$\begin{cases} \frac{dAB}{dt} = p_{ABS} \times SASC + p_{ABL} \times LASC - u_{AB} \times AB, \\ \frac{dSASC}{dt} = p_{SASC} \times I(t \leq h) - u_{SASC} \times SASC, \\ \frac{dLASC}{dt} = 0. \end{cases} \tag{4}$$

Next, presuming a proportional activation SASC, we obtain Model 5 as follows:

$$\begin{cases} \frac{dAB}{dt} = p_{ABS} \times SASC + p_{ABL} \times LASC - u_{AB} \times AB, \\ \frac{dSASC}{dt} = p_{SASC} \times SASC \times I(t \leq h) - u_{SASC} \times SASC, \\ \frac{dLASC}{dt} = 0. \end{cases} \tag{5}$$

Finally, we consider that SASC is not activated, which leads to model 6:

$$\begin{cases} \frac{dAB}{dt} = p_{ABS} \times SASC + p_{ABL} \times LASC - u_{AB} \times AB, \\ \frac{dSASC}{dt} = -u_{SASC} \times SASC, \\ \frac{dLASC}{dt} = 0. \end{cases} \tag{6}$$

Appendix B.Algorithm parameter values used in Monolix

The values utilized in the SAEM-MCMC algorithm and loglikelihood estimate in Monolix are summarized in the table below:

Population parameters	SAEM	$K_0 = 150$
		$K_1 = 500$
		$K_2 = 200$
		$\alpha_1 = 0$
		$\alpha_2 = 0.7$
	MCMC	$m_1 = 50$
		$m_2 = 10$
		$\rho = 0.05$
	Simulated annealing	$\tau_1 = 0.95$
		$\tau_2 = 0.95$
Loglikelihood	Importance sampling	Monte-Carlo size=10^7

Table 1: The values used in the SAEM-MCMC algorithm and loglikelihood estimate in Monolix.

Appendix C. Detailed model selection procedures

For model selection, ODE Model formulations were evaluated. A summary of the many models utilized to model the Herpes zoster antibody data is provided in the table below. The first column is the model identifier. In the event of convergence, the AIC value of each model is displayed in the second column. The results of the executed bootstraps are shown in the third column. The findings of probable IDs with aberrant existence in the converging bootstrap samples are shown in the fourth column. In the event that IDs are obtained, the fifth column contains the results of a bootstrap on the dataset.

Model	Convergence-AIC	Bootstrap convergence	Deviating IDs	Bootstrap w/o ID
Model 1	4266.62	68%	No	
Model 2	4619.51	No		
Model 3	4259.74	69%	Yes	70%
Model 4	4244.92	73%	No	
Model 5	4247.61	71%	No	
Model 6	4243.84	96%	No	

Table 2: ODE Model selection procedure.

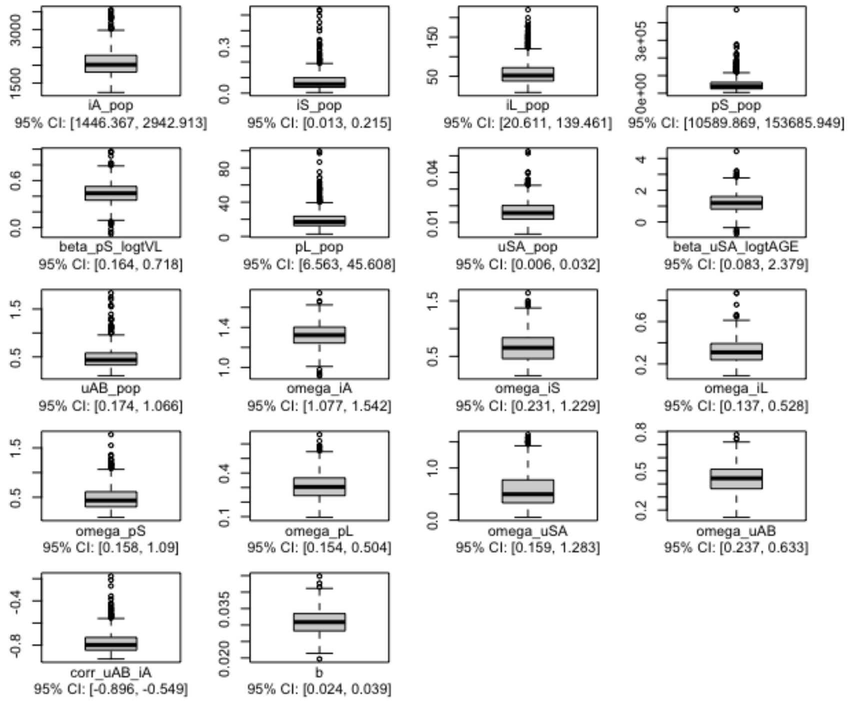

Fig 1: **Parameter estimation for model 6**

Models and convergence results are provided in the table above, results that include fixed parameters have not been added to the table as no significant findings were obtained. Model 6 has the lowest AIC value while still providing adequate bootstrap convergence (96% of the datasets had good SAEM convergence). The bootstrap that subsequently was performed did not converge for model 2, and it was therefore rejected. The existence or absence of particular individual profiles was then studied to see if the bootstrap

samples' convergence was affected. In fact, it was revealed that 69% of the bootstrap samples resulting in non-convergence for model 3 included all IDs in their dataset. As a result, a new bootstrap was performed with model 3 without the deviating ID, a new bootstrap was performed without the deviating IDs, and 71% of bootstrap samples converged. Model 6, which also demonstrated sufficient bootstrap convergence, was then considered. We investigated the relationship between the bootstrap convergence and the presence or absence of individual profiles, as we did with the previous model. Because no deviant occurrences were discovered, model 6 was chosen as the final model.

Appendix D.Numerical results for the best fitting model

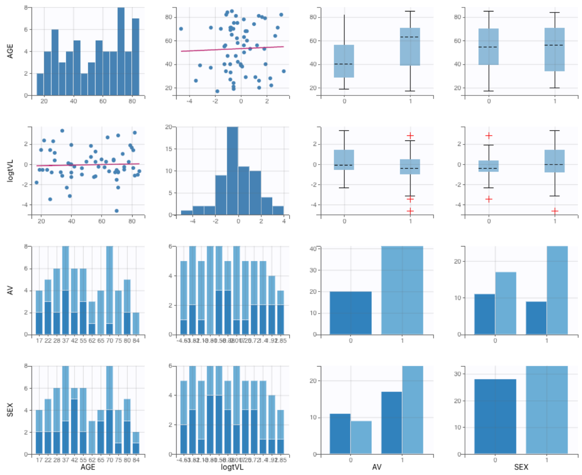

Fig 2: **The matrix of the four covariates.** Here we display the age versus the viral load (VL) and show the correlation coefficient (0,038), Sex as Categorical covariates w.r.t. other categorical covariates antiviral AV are displayed as a histogram, and continuous covariates (age and VL) w.r.t. categorical covariates (Sex and AV) are displayed as a boxplot.

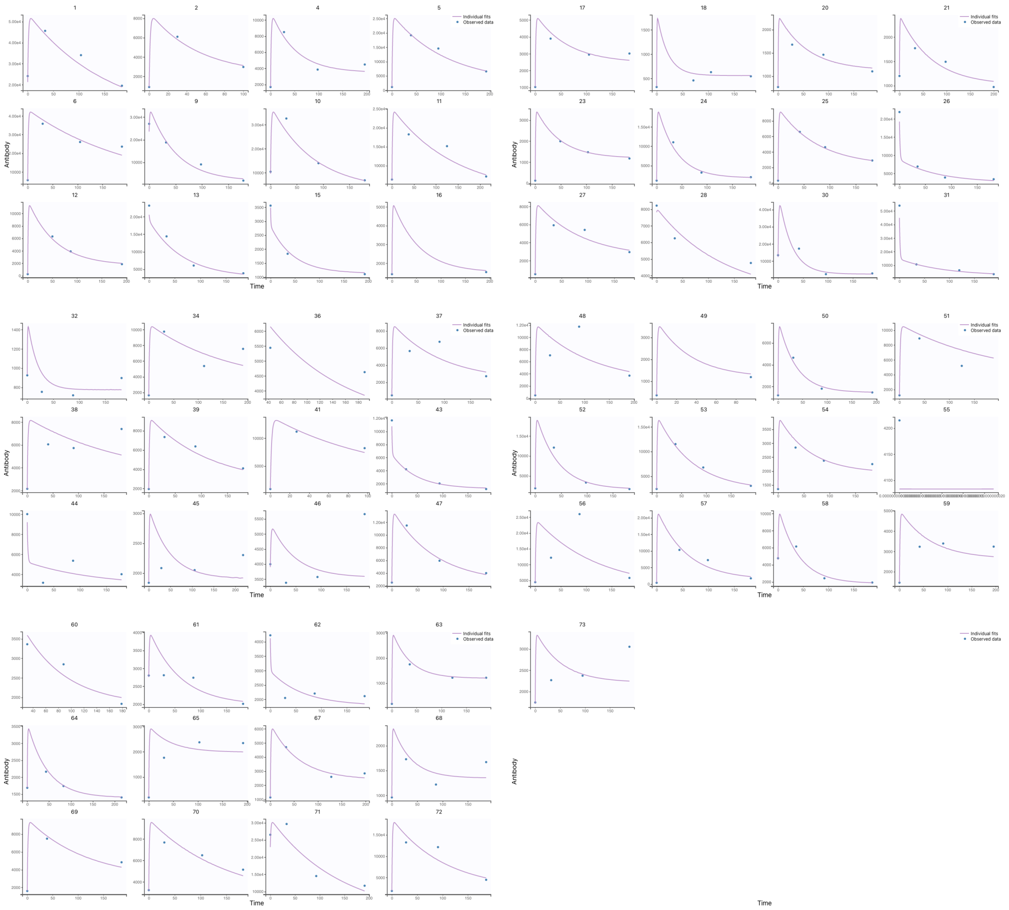

Fig 3: **Individual fits for 61 participants.** Individual predictions utilizing individual parameters and individual variables with respect to time on a continuous grid with observed data overlaid are displayed in this graphic.

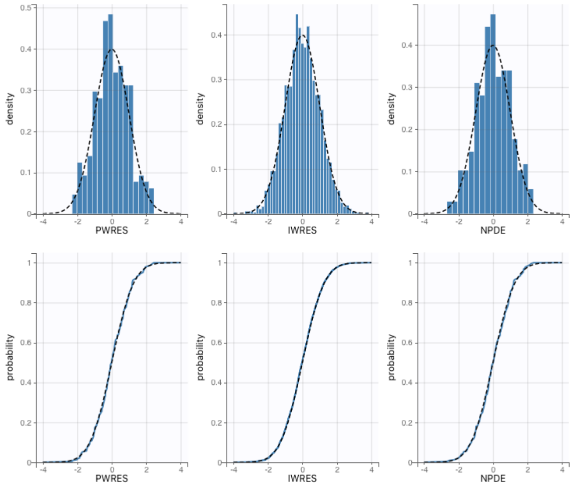

Fig 4: **Distribution of the residuals.** PWRES, IWRES, and NPDE distributions are shown as histograms for the probability density function (PDF) and as cumulative distribution functions in this graphic (CDF).

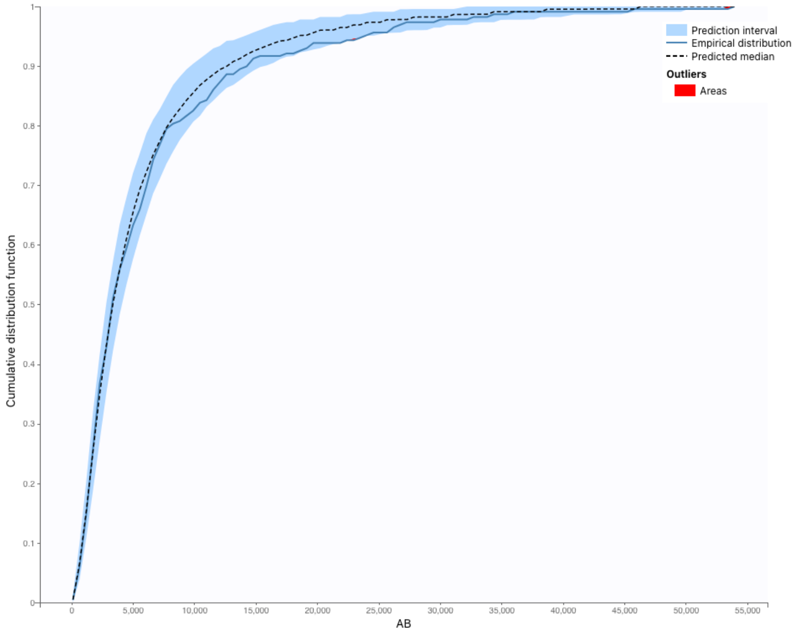

Fig 5: **Numerical predictive check (NPC).** The NPC is a model diagnosis tool for continuous data that allows you to compare the empirical cumulative distribution function (CDF) of the observations, which was computed on the original data set, with the theoretical cumulative distribution, which was computed from data simulated with the model 6 and the original data set's design structure. (There is no red area in the plot meaning no Outliers)

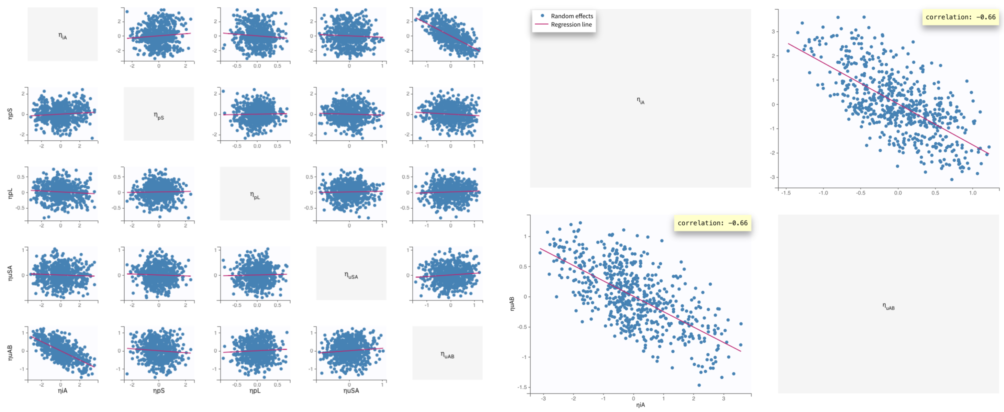

Fig 6: **Correlation between random effects.** This graph shows scatter plots for each random effect pair. It can be used to find correlations between random effects, such as the one between AB_0 and u_{AB} with a Pearson correlation coefficients of 0.66 in addition to regression lines.

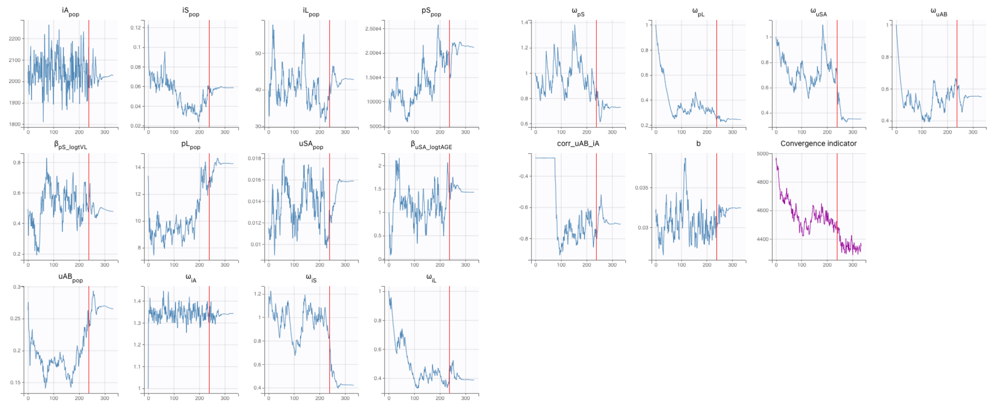

Fig 7: **Convergence of SAEM.**The sequence of population parameter estimates calculated after each iteration of the SAEM algorithm is shown in this graph. The goal is to see if the algorithm is convergent. In addition, a convergence indicator calculates the -2 x log-likelihood estimate for each iteration.The vertical line indicates where the algorithm switches from the Exploratory phase to the Smoothing phase. The convergence indicator in purple decreases progressively and then stabilizes,which mean that the maximum likelihood has been achieved.

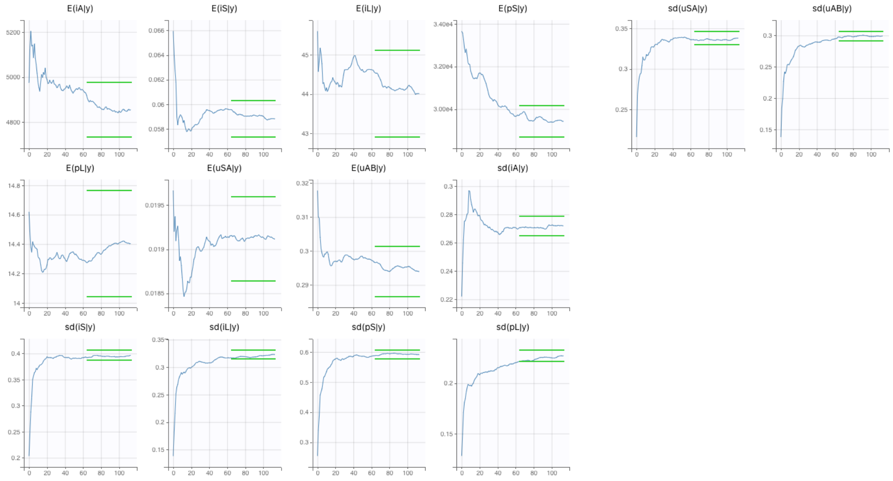

Fig 8: **Convergence of MCMC.** During individual parameter estimation by Markov Chain Monte Carlo, this plot shows the sequence of estimates for the conditional means and conditional standard deviations along the iterations of the MH algorithm. The purpose is to check the MCMC algorithm's convergence, which is clearly achieved.

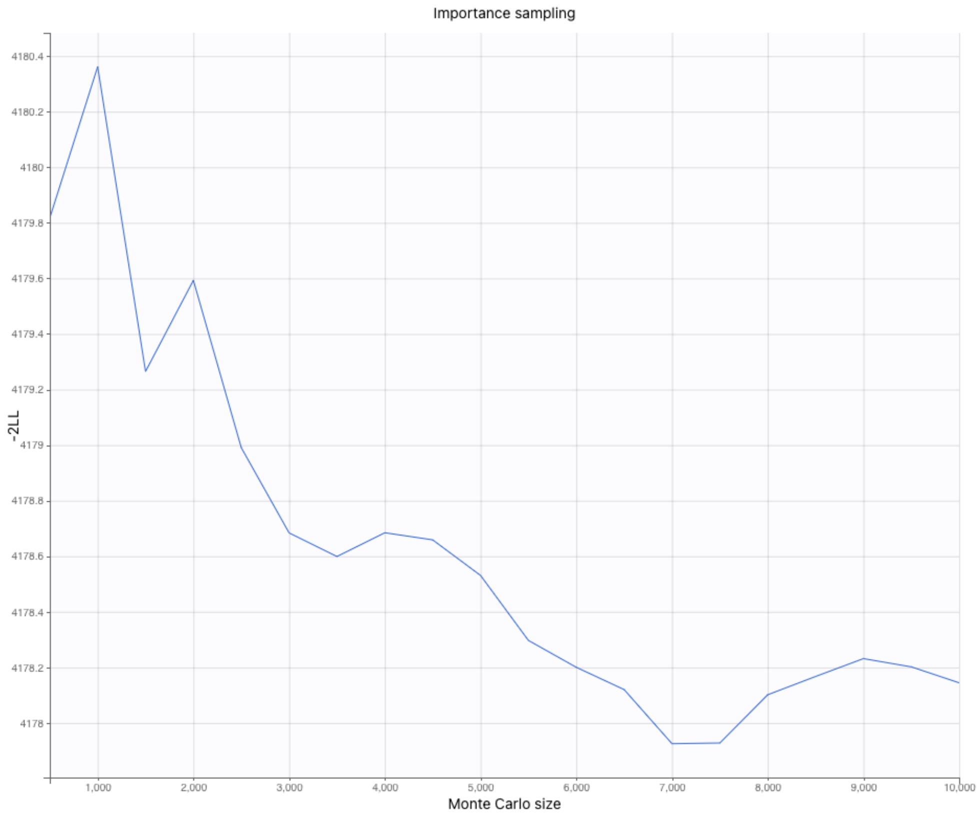

Fig 9: **Convergence of Importance sampling.** The sequence of estimates for observed log-likelihood derived using the Monte Carlo method is shown in this graph. The bias of the log-likelihood estimator reduces with the number of repetitions before the estimation value stabilizes. The number of points in the plot is usually smaller than the number of iterations, and depends on the total number of observations.
